# Screening paediatric rectal forms of azithromycin as an alternative to oral or injectable treatment

**DOI:** 10.1016/j.ijpharm.2012.07.030

**Published:** 2012-10-15

**Authors:** Tina Kauss, Karen Gaudin, Alexandra Gaubert, Boubakar Ba, Serena Tagliaferri, Fawaz Fawaz, Jean-Louis Fabre, Jean-Michel Boiron, Xavier Lafarge, Nicholas J. White, Piero L. Olliaro, Pascal Millet

**Affiliations:** a Univ. Bordeaux, EA 4575 Analytical and Pharmaceutical Developments Applied to Neglected Diseases and Counterfeits, Bordeaux, France; b Accelera s. r. l., Nerviano (MI), Italy; c OTECI (Office Technique d’Etude et de Coopération Internationale), Paris, France; d EFS (Etablissement Français du Sang) Aquitaine Limousin, Bordeaux, France; e Faculty of Tropical Medicine, Mahidol University, Bangkok, Thailand; f Centre for Tropical Medicine, Nuffield Department of Medicine, University of Oxford, UK; g UNICEF/UNDP/WB/WHO Special Program for Research & Training in Tropical Diseases (TDR), Geneva, Switzerland

**Keywords:** Azithromycin, Antibiotic, Rectal, Formulation, Bioavailability

## Abstract

The aim of this study was to identify a candidate formulation for further development of a home or near-home administrable paediatric rectal form of a broad-spectrum antibiotic – specially intended for (emergency) use in tropical rural settings, in particular for children who cannot take medications orally and far from health facilities where injectable treatments can be given. Azithromycin, a broad-spectrum macrolide used orally or intravenously for the treatment of respiratory tract, skin and soft tissue infections, was selected because of its pharmacokinetic and therapeutic properties. Azithromycin *in vitro* solubility and stability in physiologically relevant conditions were studied. Various pharmaceutical forms, *i.e.* rectal suspension, two different rectal gels, polyethylene glycol (PEG) suppository and hard gelatin capsule (HGC) were assessed for *in vitro* dissolution and *in vivo* bioavailability in the rabbit. Azithromycin PEG suppository appears to be a promising candidate.

## Graphical abstract

**Figure:**
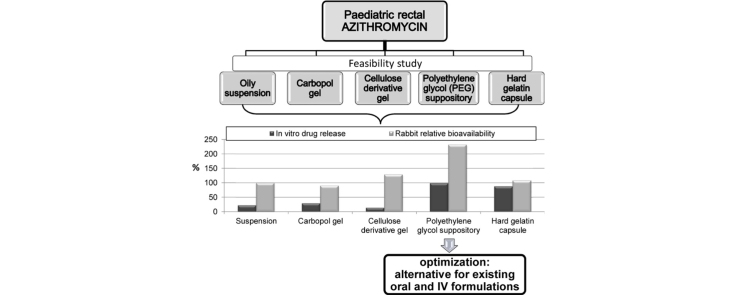

## 1 Introduction

Antibiotics are generally available in oral or parenteral formulations. A limitation of either route is that they may be unsuited in children, especially those who cannot take oral medications and/or live in environments where injections may not be immediately available or would be unsafe – as it is often the case in rural areas in developing countries with limited access to health facilities. In these conditions, the rectal route could be a valuable alternative (Ernest et al., 2007). The rectal route is non-invasive easy-to-use and can be applied to patients who are unable or unwilling to take medications orally. Besides, it avoids at least partially the first pass effect, improves the stability of drugs undergoing gastro-intestinal degradation, and allows the administration of unpleasantly tasting or smelling drugs (Allen, 2008). Main compounding and manufacturing formulas of suppository formulations have been compiled in Pharmaceutical Press's recent edition on suppository (Allen, 2008) and investigated antibiotic suppository formulations reviewed (Bergogne-Bérézin and Bryskier, 1999). There are examples like malaria where pre-referral rectal administration is feasible and saves lives (Gomes et al., 2009). Bacterial infections like pneumonia and meningitis take a heavy toll on children in the tropics, particularly those under five years of age, and together cause over one thousand DALYs (disability-adjusted life years, a measure of potential future years lost due to poor health), which is comparatively more than HIV. The World Health Organization (WHO) estimates that globally 363,000 and 735,000 HIV-negative children under five years of age died in 2000 due to *Haemophilus influenzae* type b and *Streptococcus pneumoniae* infections, respectively. In addition, an estimated 50 million cases of *Bordetella pertussis* and 300,000 deaths occur every year; case-fatality rates in developing countries are estimated by the WHO to be as high as 4% in infants (WHO, 2012). These infections are prevalent in rural settings of the tropical world, with poor access to health facilities – meaning that diagnosis and treatment may be delayed, putting children lives in danger. Children who become progressively unwell will have no treatment in the vicinity, as they will no longer be able to take oral medications, while injections may be hours away as they are not available outside medical facilities. This is when rectal formulations may save lives.

In order to be used in rural settings, a product must be not only effective and safe, but also affordable and stable in tropical climate. A possible candidate is azithromycin, a semi synthetic acid-stable macrolide with an expanded spectrum and improved pharmacokinetic characteristics over older macrolides (Dunn and Barradell, 1996), thus allowing short, once daily regimens (Langtry and Balfour, 1998). Structurally related to erythromycin, it acts by inhibiting protein synthesis by binding to the 50S subunit of the bacterial ribosome, and covers Gram-positive and Gram-negative bacteria (Champney and Miller, 2002; Peters et al., 1992; Swainston Harrison and Keam, 2007). Its primary indications include *Chlamydia* and *Mycoplasma* infections, whooping cough (*Bordetella pertussis*) and Legionnaire's disease and used instead of penicillin in beta-lactam allergic individuals. Azithromycin is used frequently for the treatment of community-acquired acute respiratory infections. It appears to be more active against *Haemophilus influenzae* than erythromycin and against some enteric Gram-negative bacilli, although not generally indicated for the treatment of *Enterobacteriaceae*.

Furthermore, mass oral azithromycin distribution is a key component of the WHO's trachoma elimination program and has been associated with a reduction in all-cause mortality (Coles et al., 2012; Porco et al., 2009; WHO, 2012). Azithromycin spectrum of activity covers most bacteria responsible for the main causes of infants and children morbidity and mortality. Pneumococcus remains susceptible to azithromycin in most areas of Sub-Saharan Africa and in Asia. Azithromycin also proves effective against *Salmonellae* spp. in Asia (Ashley et al., 2011; Buchanan et al., 2010; Lubell et al., 2011).

Azithromycin is generally administered at 30 mg/kg over 3 (3 × 10 mg/kg/day) or 5 (10 mg/kg/day + 4 × 5 mg/kg/day) days. A single-dose regimen of azithromycin has recently been proposed using controlled release tablets (Gandhi et al., 2004) or extended release microspheres (Lo et al., 2009; Swainston Harrison and Keam, 2007). This regimen has been shown to be as effective as the same dose administered over several days in experimental animal models (Girard et al., 2005). Currently, single administration of 30 mg/kg azithromycin in paediatric patients with acute otitis media is approved in the USA (Pfizer, 2012).

Azithromycin absorption after oral administration is relatively rapid, inducing in 2 h a peak plasma concentration (*C*_max_) of 383 μg/l and 224 μg/l in children from 0.6 to 5 years and 6 to 15 years respectively after 5 day regimen (10 mg/kg + 4 days 5 mg/kg). (Langtry and Balfour, 1998). The drug distributes well to infected fluids, tissues and intracellularly, where therapeutically-relevant concentrations are maintained long after plasma concentrations have diminished. The elimination half-life in children is 32 to 64 h and total body clearance is 4.5 to 5.4 l/h/kg, offering the convenience of a short, once-daily regimen (Langtry and Balfour, 1998). In adults, the absolute bioavailability of azithromycin 250 mg oral capsules has been estimated at 38% of the intravenous dose (Pfizer, 2012).

While erythromycin suppositories have been studied in paediatric patients (though no commercially-available product exists today to our knowledge), there is only one published study for azithromycin reporting measurable but low (3.2%) rectal absorption (Bergogne-Bérézin and Bryskier, 1999). For erythromycin, rectal bioavailability (28–54%) was found to depend on the age of children (Stratchunsky et al., 1991)

The aim of this project was to identify a suitable formulation of azithromycin that would produce adequate plasma levels, cover the main paediatric bacterial infections, and be affordable and acceptable for use in tropical countries. These requirements limited us to conventional liquid, solid or semi-solid forms. This article describes the pharmaceutical screening of four different azithromycin rectal forms, two hydrogels, a carbopol gel and hydroxypropyl methyl cellulose (HPMC) gel, hard gelatin capsule and a PEG suppository, compared to intra-venous and rectal controls. For this purpose several azithromycin rectal formulations were developed and evaluated for their bioavailability in rabbits.

## 2 Material and methods

### 2.1 Material

Azithromycin dihydrate was a gift from Pfizer, France. Zithromax^®^ (Pfizer) was used as IV azithromycin formulation. For all our experiments, commercially available azithromycin dihydrate was chosen, as the most stable form of azithromycin hydrates (Gandhi et al., 2002).

Pharmaceutical excipients, namely PEG 1500 and PEG 4000 (Fagron, France), hydroxypropylmethylcellulose (HPMC) 4000 (Colorcon, Hollande), microcrystalline cellulose (Emcocel 90, Cooper, France), colloidal silica (Aerosil 300, Cooper, France), carbopol 974P NF (Noveon, OH), triethylamine (Sigma, France), propylene glycol (Cooper, France), miglyol 812N (Inresa, France), citric acid, NaOH and NaCl were of pharmaceutical grade. Chemicals used for analysis (acetonitrile, ammonium formate, phosphate buffer) were of analytical grade.

### 2.2 Azithromycin solubility and stability evaluation in *in vitro* simulated rectal pH conditions

#### 2.2.1 Azithromycin apparent solubility in 4 h at pH 7–9

An excess of azithromycin (about 250 mg precisely weighted) was introduced into 5 ml of 50 mM phosphate buffer of pH 7.0, 8.0 or 9.0. After 4 h of magnetical stirring (600 rpm) at room temperature in hermetically closed recipients, samples were filtered with nylon 0.22 μm syringe filters and adequately diluted in mobile phase before HPLC analysis.

#### 2.2.2 Azithromycin solution stability at pH 7–9

200 ml of extemporaneous azithromycin solution was prepared in 50 mM phosphate buffer pH 7.0, 8.0 or 9.0. After complete dissolution of azithromycin (room temperature ultrasounds 20 min) the solutions were filtered through syringe nylon filters of 0.45 μm. At time *T*0, samples (*n* = 3, 10 ml) were taken for *T*0 analysis and hermetically closed vials containing the rest of solutions were placed at 37.0 °C. Further samples (*n* = 3, 10 ml) were taken at *T*0 + 4 h, *T*0 + 8 h and *T*0 + 24 h and analysed using HPLC (as shown in Section 2.3.2).

### 2.3 Azithromycin pharmaceutical formulations

#### 2.3.1 Preparation of azithromycin formulations

All formulations tested except IV (Zithromax^®^, Pfizer) were developed in our laboratory. The formulations were optimized for their pharmacotechnical properties, compatibility with excipients used and drug release properties. Table 1 summarizes final formulations considered.

Rectal suspension (formulation A) was prepared by dispersion of azithromycin (sieved at <250 μm) in medium chain triglyceride oil (miglyol 812N). Carbopol gel (formulation B) was prepared by dispersion of azithromycin (<250 μm) and then Carbopol^®^ in propylene glycol/water mixture and neutralized using triethylamine. HPMC gel (formulation C) was prepared using HPMC as mucoadhesive gelling excipient, by dispersion of azithromycin (<250 μm) and then HPMC in water/ethanol mixture. Azithromycin suspended suppositories (formulation D) were prepared by dispersion of azithromycin (<250 μm) in melted PEG mixture at 70 °C (water bath) and cooled at room temperature. All dispersions were performed under electrical stirring, which was maintained during sampling for liquid and semi-liquid preparations. Azithromycin HGC were prepared using Turbula^®^ (Erweka, France) powder blender and semi-automatic HGC filler and loader LGA QB 300 (LGA, France). All forms were prepared extemporaneously.

Pharmaceutical forms used for *in vivo* evaluation in rabbits were adjusted to animal weight and administered using 1 ml syringe for liquid and semi-liquid formulations and 1 ml pipette like device for dry forms.

In case of suppositories, the same formulation was administered to animals at 20 mg/kg and 40 mg/kg. All other tested formulations were administered at 20 mg/kg. Zithromax^®^ at 10 mg/kg was used as IV reference.

#### 2.3.2 *In vitro* evaluation of azithromycin formulations

Visual homogeneity, mean assay (*n* = 3) and dissolution tests (*n* = 6) were performed for all rectal formulations.

For azithromycin content, approximately 450 mg of formulation was precisely weighted and completed up to 20 ml with HPLC mobile phase. After 60 min of magnetical stirring at 600 rpm, the preparation was filtered at 0.22 μm nylon syringe filters before analysis.

Dissolution tests (*n* = 6 for each formulation) were performed in European pharmacopoeia apparatus 2 containing 250 ml of phosphate 50 mM buffer pH 7.0 per bowl at 37.0 °C and 75 rpm. Rectal liquid or semi-liquid formulations (A–C, approximately 6 g precisely weighted formulation) were tested in dialysis membrane bags (Spectra/Por^®^ molecular porous membrane, diameter 25 mm, MWCO 12-14.000, Spectrum Laboratories, USA) and ballasted to assure their immersion. Samples (1 ml, replaced with fresh dissolution medium) were taken at *T*0 and 15/30/45/60/90/120 min with 10 μm nylon pre filter and injected directly on HPLC system.

#### 2.3.3 HPLC analysis of azithromycin *in vitro* samples

HPLC analyses were performed on Waters HPLC system with 515 HPLC Pump, 717plus Autosampler and 2487 Dual λ Absorbance Detector (Waters, France). Previously described HPLC method for azithromycin determination was used (Gaudin et al., 2011). Briefly, a mixture methanol/phosphate buffer 15 mM (80:20, v/v) with apparent pH of 9 was used as a mobile phase and filtered on nylon 0.22 μm membrane filter before use. UV detection was set at 210 nm and the flow rate was set at 1 ml min^−1^. 10 μl of each sample was injected. The column used was Luna C8 EC 5 μm, 150 mm × 4.6 mm (Phenomenex, France) thermostated with Crococil oven (CIL, Saint Foy la Grande, France) at 20 °C.

### 2.4 Animal pharmacokinetic experimentation

Adult New Zealand White KBL rabbits were used for pharmacokinetic studies after at least 10 days of acclimation. Animals were placed into individual cages in controlled temperature (17–21 °C) and humidity (40–70%) room. They were fastened 12 h before starting pharmacokinetic study, but had water ad libitum.

The administration of each formulation was adapted individually to the rabbit body weight.

Blood samples (about 0.5 ml of blood per time point) were collected from peripheral ear vessels into heparinized plastic tubes (opposite ear in case of IV administration), kept on an ice-water bath, and then centrifuged (10 min, 1200 g, +4 °C). At least 200 μl of plasma was stored in a freezer at −80 °C until analysis. Blood samples were collected at pre-dose, then 6 samples in 24 h post-administration according to formulations.

Groups of three or four animals were used for pharmacokinetic screening of azithromycin formulations for suppositories and other formulations respectively.

Pharmacokinetic parameters, namely *C*_max_, *T*_max_ and AUC of each condition were obtained from each individual plasma profile and then mean and standard deviation (SD) were calculated. AUC was calculated using trapezoid rule.

### 2.5 Pharmacokinetic sample preparation and LC–MS/MS analysis conditions

Plasma proteins were precipitated by adding 200 μl of methanol to 10 μl of plasma in a 96 well plate. After capping and vortex mixing, the plate was centrifuged for 15 min at 2060 × *g* at 6 °C. An aliquot of 150 μl of supernatant was transferred into a new 96 well plate and dried at 40 °C. Sample extract was reconstituted with 150 μl of a mixture solution of 10 mM ammonium formate pH 3.5/acetonitrile (50:50, v/v). After capping and vortex mixing, the plate was centrifuged for 3 min at 2060 × *g* at 6 °C and the supernatant was injected onto the LC–MS/MS system. Standards were made with rabbit plasma using heparin as anticoagulant. 10 μl of each sample was injected.

HPLC system was Hewlett Packard 1100 series equipped with autosampler CTC Analytics CTC PAL refrigerated at 10 °C. The separation was performed with Zorbax SB-C8 4.6 mm × 75 mm, 3.5 μm column. The mobile phase used was a gradient mixture of 10 mM ammonium formate pH 3.5 and acetonitrile (45/55%, v/v at 0–1.7 min, 30/70% (v/v) at 1.8–2.5 min, 10/90% (v/v) at 2.6–3.5 min and 45/55% (v/v) at 3.6–5 min) with a total run of 5 min. The flow rate was set to 1.0 ml min^−1^ and the column oven temperature to 40 °C. In these conditions, the approximate retention time of azithromycin was 2.02 min.

MS instrument used was Perkin Elmer SCIEX API 3000 with turbo ion spray ionization in positive ion mode. Azithromycin fragments for MRM transitions were *m/z* 375.25 and *m/z* 591.30. The lower and the upper quantification limits were determined to be 1.00 and 2060 ng/ml respectively.

### 2.6 Statistical analysis of data

*In vitro* obtained data were analysed using Student's bilateral paired *t* test. The difference is considered significant for *p* < 0.05.

For *in vivo* data, the reduced number of experimental animals rendered usual statistical biopharmaceutical (bioequivalence) analysis like ANOVA not appropriate. Mean ± SD values were given for each result.

## 3 Results and discussion

### 3.1 Dissolution and stability of azithromycin in physiologically-relevant conditions

The dissolution of azithromycin hydrates is reportedly slow (Gandhi et al., 2002), requiring 48 h to reach equilibrium solubility in water under 100 rpm stirring. For rectal administration, azithromycin *in situ* dissolution was considered as a potential absorption-limiting factor. We studied azithromycin apparent solubility over clinically-relevant conditions for duration, temperature and pH range. The rectal pH is reportedly near-neutral in healthy and unwell infants >28 days old (range 6.68–7.12) (Turner et al., 2011), though more alkaline values (7.2–12.1) were found in one study in children (Jantzen et al., 1989).

**Fig. 1:**
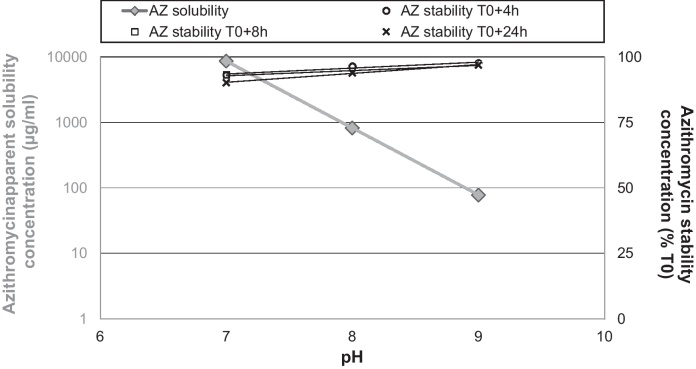
Variation within rectal physiological pH range of azithromycin apparent solubility concentrations after 4 h of stirring (left, *Y* axis) and stability of azithromycin concentrations after 4, 8 and 24 h at 37 °C after dissolution (right, *Y* axis). Legend: AZ = azithromycin.

After incubation at 37 °C for 4 h in phosphate buffer at pH 7–9 a log-linear decrease in azithromycin solubility was found with increasing pH (Fig. 1). This is consistent with azithromycin p*K*a values (8.7 and 9.5) (Zhang et al., 2009): when pH < p*K*a, azithromycin is protonated and consequently more soluble in water.

The second experiment aimed at verifying whether once dissolved, azithromycin remained stable. The experiment was extended to 24 h so as to exceed the usual residence time in rectum. The azithromycin solution stability was better at basic than neutral pH. Azithromycin content (Fig. 1) remained >90% throughout the 24 h in all tested pH conditions (7–9) at 37 °C. Previous studies have shown that at pH 8, the half-life of azithromycin in buffered solution was 54 days at 20 °C and 6 days at 40 °C (Esteban et al., 2009).

The relative stability and apparent solubility performances of azithromycin were deemed compatible with rectal administration.

### 3.2 Pharmaceutical development and *in vitro* evaluation

Several pharmaceutical forms, namely rectal gels, hard gelatin capsules and suppositories, were developed for the purpose (Table 1). The spectrum of pharmaceutical forms was restricted by our target product profile, to opt that it would be inexpensive, could withstand tropical conditions, is easy to use by children and delivered by untrained persons. Thus, novel technologies, e.g. nano- and microtechnologies were not considered, as they would be unaffordable. A series of assays was performed to ensure the feasibility and obtain correct pharmaco-technical performances. A muco-adhesive gel was tested in order to enhance rectal bioavailability by increasing the residence time *in situ* as compared to classical non muco-adhesive formulations. The content of the gelling agent (carbopol 0.6–1.0% (w/w) or HPMC 2.0–2.8% (w/w)) was adjusted to adapt the thickness of the formulation to a possible administration with a syringe. Soluble suppositories, based on PEG mixtures, were preferred to fatty fusible suppositories as they have a higher melting point, thus not requiring special storage conditions. Previous studies showed that the melting point increases and drug release rate decreases with increasing PEG molecular weight (Leuner and Dressman, 2000). The mixture of PEG used (1500 and 4000) provided for both a melting point >45 °C and relatively rapid drug release.

The HGC formulation was modified in terms of flowability of the powder mixture and in terms of homogeneity of the mixing process by adding a colouring agent in feasibility batches. Colloidal silica improved the flowability whereas talc and magnesium stearate did not. The minimal quantity of colloidal silica obtaining satisfactory flowability results was chosen for further evaluations. As reference products we used a commercially available intra-venous formulation (Zithromax^®^, Pfizer) and an oily rectal suspension. Final formulations are summarized in Table 1.

The results of *in vitro* drug release tests of the final formulations are summarized in Table 2; azithromycin content was within 100 ± 5% for all formulations, while drug release after 45 and 90 min varied according to the formulation. Rapid drug release was observed for azithromycin PEG suppositories and azithromycin HGC. The drug is released from PEG suppositories as a consequence of the progressive dissolution of PEG in the surrounding liquid. PEGs are known to increase both, drug dissolution and its solubility, explaining enhanced drug release (Allen, 2008). For HGC, the direct dissolution of small AZ particles (<250 μm) with increased contact surface allowed rapid drug release. Azithromycin HGC released drug even more rapidly than PEG suppository (*p* < 0.01, Student's *t* test). Drug release studies of gels and oily suspension of azithromycin, contrary to HCG and suppositories, required the use of dialysis bags, making results not directly comparable. Of note, azithromycin suspension in dialysis bags gave comparable results (20–30% in 90 min) as others available in the literature for free drug (Arora et al., 2010). Carbopol azithromycin gel released significantly more azithromycin (*p* < 0.05, Student's *t* test) than control oily suspension at 45 and 90 min. Drug release from HPMC gel was sustained compared to carbopol gel and statistically not different from the control suspension (*p* > 0.10, Student's *t* test). Overall, the rate of drug release was low from gels (viscosity reducing the dissolution rate and hampering the diffusion of dissolved azithromycin fractions towards dissolution medium) and oily suspension (indirect mechanism of release: dissolution in oily phase, diffusion towards interface, partition between aqueous and oily medium) (Table 2).

The long term ICH stability studies were outside the scope of our preliminary project, and are planned only for the final, biopharmaceutically optimized formulation. Nevertheless, suppositories and hard gelatine capsules were kept in ambient condition in plastic moulds or closed glass bottle respectively. Samples were periodically analysed by HPLC for their drug content. Suppositories were stable (95–105% AZ content) after 2 years and hard gelatine capsules after 6 months (end of study). In view of *in vitro* and *in vivo* drug release data, gel's stability was not analysed further.

### 3.3 *In vivo* bioavailability screening of azithromycin formulations

**Fig. 2:**
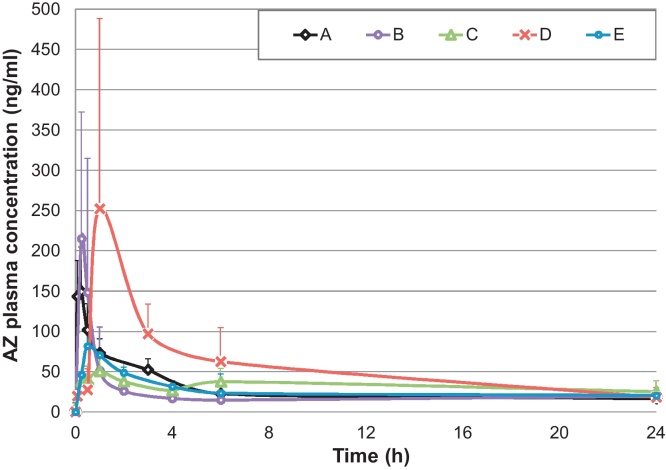
Azithromycin rabbit plasma concentration profiles after rectal administration of 20 mg/kg azithromycin in various formulations. Legend: A = azithromycin rectal suspension (rectal reference); B = azithromycin carbopol gel; C = azithromycin mucoadhesive gel; D = azithromycin suppository, E = azithromycin HGC.

Azithromycin rectal formulations were tested in rabbits at 20 mg/kg and compared to IV (10 mg/kg) and to a fatty suspension of azithromycin (formulation A, 20 mg/kg) used as rectal route reference. Doses were determined based on previously published animal pharmacokinetic data (Shepard and Falkner, 1990). Fig. 2 shows rabbit plasma profiles of rectally administered formulations and Table 3 summarizes the main pharmacokinetic parameters.

Azithromycin rectal absorption was rapid for the suspension and carbopol gel (*T*_max_ 15 min), while it required 30 min to reach maximum plasma concentration for the HGC and 60 min for the HPMC gel and the PEG suppository. These results are in line with *in vitro* dissolution data. The highest rectal *C*_max_ and rectal AUC_0−24 h_ was obtained with the PEG suppository; relative bioavailability was ∼28% of IV and ∼2.3 times that of the rectal suspension. All other formulations produced similar exposures between 11–16% of IV and 90–129% of rectal suspension (as shown in AUC_0−24 h_, Table 3).

The ratios AUC_24 h_/minimum inhibitory concentration (MIC) and *C*_max_/MIC are regarded as the main pharmacokinetic/pharmacodynamic parameters for azithromycin efficacy. Differently from erythromycin, for azithromycin the AUC_24 h_/MIC is more important than T/MIC because of its long half-life and good tissue penetration (Van Bambeke and Tulkens, 2001). Of the formulations tested, the PEG suppository appears to be the most promising candidate, but has a potential shortcoming – the rather long *T*_max_, which is of particular concern if early onset of action is needed.

The suspended PEG suppository is a prototype and will require further, biopharmaceutical optimization work. The 28% bioavailability relative to IV is an encouraging result compared to 38% for the existing oral formulation (extrapolated 75% relative bioavailability of suppositories compared to oral capsules). While values in humans cannot be directly inferred from (limited) animal data, our results are within the range of values described in the literature in terms of *C*_max_, namely 2400 ng/ml after 10 mg/kg azithromycin IV dose in children (Jacobs et al., 2005), 400–500 ng/ml after 500 mg azithromycin oral dose in adults (Boonleang et al., 2007; Gandhi et al., 2004) and 318 ng/ml after 12 mg/kg oral dose in children (Stevens et al., 1997). The bioavailability of the suppository formulation could be further improved by adding absorption enhancers or using a eutectic mixture of PEG and azithromycin. The principles used to improve drug solubility for oral delivery (Shepard and Falkner, 1990) could be applied to rectal formulations.

It should be noted that, due to the ethical requirement to minimize the number of animal tested and the relatively high concentrations still persisting 24 h post-dosage (*C*_24 h_ ≈ 1/10*C*_max_ except for HPMC gel *C*_24 h_ > 1/10*C*_max_), it was not possible to estimate correctly AUC_0–∞_.

Even though rectal tolerance studies were beyond the scope of the current screening work, no significant change in rabbit behaviour was evidenced that could be ascribed to tolerability issues, nor was the presence of blood at the anal sphincter with any of the formulations.

### 3.4 Dose-dependence of azithromycin PEG suppositories

The effect of azithromycin dose on exposure is reported in Table 4. Pharmacokinetic parameters did not vary proportionally with the dose of azithromycin administered. *C*_max_ remained unchanged for 20 and 40 mg/kg of azithromycin (261 and 255 ng/ml respectively) and the extrapolated AUC value ratio was 1:1.5 compared to 1:2 ratio for the azithromycin dose. *T*_max_ remained unchanged.

Again, caution should be applied when interpreting these results, both because of the small number of experimental animals and because plasma concentrations may not reflect the true total exposure in peripheral compartments. While both plasma and tissue concentrations were reportedly increased when doubling azithromycin dose from 500 to 1000 mg per os in healthy adults (Danesi et al., 2003), an oral dose of 20 mg/kg produced only a marginal increase in plasma AUC over 10 mg/kg (AUC ratio 1:18), but a more significant increase of tonsil tissue AUC (AUC ratio 1:53) (Blandizzi et al., 2002). Future studies with improved formulations should allow extended sampling time and ideally measure tissue levels as well as plasma concentrations.

## 4 Conclusion

An azithromycin suspended PEG suppository was identified as a promising candidate for further pharmaceutical development with the aim of achieving comparable bioavailability to the oral route. Such formulation would be an easy-to-use, safe and cheap alternative to oral and injectable forms. While the reason for developing a rectal form was primarily intended for near-home emergency use in rural settings of developing countries, a PEG suppository of a broad-spectrum antibiotic would also be a useful addition to the current paediatric therapeutic armory.

## Figures and Tables

**Table 1:** Galenic rectal formulations of azithromycin.

	Formulation				
	A	B	C	D	E
Pharmaceutical form	Suspension	Carbopol gel	HPMC gel	Suppository	Hard gelatin capsule
Dose (mg/kg)	20	20	20	20	20
Dosage by	Volume	Mass	Mass	Mass	Mass
Composition unit	% (w/v)	% (w/w)	% (w/w)	% (w/w)	% (w/v)
Azithromycin 2H_2_O^a^	2.1	2.1	2.1	16.2	80.48
Carbopol 974P NF	–	0.8	–	–	–
Triethylamine	–	0.8	–	–	–
HPMC 4000	–	–	2.4	–	–
Microcrystalline cellulose	–	–	–	–	19.21
Colloidal silica	–	–	–	–	0.31
PEG 1500	–	–	–	16.9	–
PEG 4000	–	–	–	67.7	–
Propylene glycol	–	84.0	–	–	–
Miglyol 812N	97.9	–	–	–	–
Ethanol (99%)	–	–	10.0	–	–
Water	–	12.3	85.5	–	–

^a^2.1 g and 16.2 g of azithromycin 2H_2_O is equivalent to 2.0 g and 15.4 g of anhydrous azithromycin respectively.

**Table 2:** *In vitro* evaluation of azithromycin rectal formulations.

	Azithromycin formulation				
	A	B	C	D	E
Azithromycin content (mean ± SD, *n *= 3)	100.8 ± 4.1	102.9 ± 2.3	99.5 ± 2.0	98.3 ± 0.9	100.3 ± 1.2
Drug release at 45 min (mean ± SD, *n *= 6)	5.5 ± 0.8	13.4 ± 7.9*	5.5 ± 0.5	73.8 ± 1.8**	88.0 ± 16.4**
Drug release at 90 min (mean ± SD, *n *= 6)	22.8 ± 10.3	29.5 ± 19.4	14.0 ± 6.0	98.9 ± 1.4**	88.3 ± 9.5**

Legend: A = azithromycin rectal suspension (rectal reference); B = azithromycin carbopol gel; C = azithromycin HPMC gel; D = azithromycin suppository, E = azithromycin HGC; *p < 0.05 Student's *t* test compared to formulation A; **p < 0.01 Student's *t* test compared to formulation A.

**Table 3:** Pharmacokinetic parameters of various azithromycin formulations (IV, A–E) in rabbits (mean ± SD).

Pharmacokinetic parameter	Formulation					
	IV	A	B	C	D	E
*C*_max_ (ng/ml)	2251 ± 696	171 ± 37	215 ± 157	59 ± 11	261 ± 228	89 ± 11
*T*_max_ (h)	0.08 ± 0.00	0.50 ± 0.31	0.25 ± 0.00	3.50 ± 2.50	1.67 ± 0.94	0.83 ± 0.12
AUC_0–24 h_ (ng h/ml)	2475 ± 648	603 ± 171	546 ± 308	779 ± 107	1400 ± 919	646 ± 293
(AUC_0–24 h_ × IV dose)/ (AUC_0–24 h__IV_ × dose x)	100	12.2	11.0	15.7	28.3	13.1
(AUC_0–24 h_ × dose A)/ (AUC_0–24 h__A_ × dose x)	–	100	90.6	129.1	231.9	107.1

Legend: A = azithromycin rectal suspension (rectal reference); B = azithromycin carbopol gel; C = azithromycin HPMC gel; D = azithromycin suppository, E = azithromycin HGC; x = studied formulation (IV or A–E).

**Table 4:** Comparison of pharmacokinetic parameters of 20 mg/kg (*D*_20_) and 40 mg/kg (*D*_40_) azithromycin suppository administration in rabbits (mean ± SD *n* = 3).

Formulation	*D*_20_	*D*_40_	*D*_40_/*D*_20_ ratio
Azithromycin dose (mg/kg)	20	40	2
*C*_max_ (ng/ml)	261 ± 228	255 ± 203	1.0
*C*_max_/Dose (ng/ml/mg/kg)	13.1	6.4	0.5
*T*_max_ (h)	1.67 ± 0.94	1.67 ± 0.94	1
AUC_0–24 h_ (ng h/ml)	1400 ± 919	2026 ± 1328	1.5
